# Multisensor Capacitance Probes for Simultaneously Monitoring Rice Field Soil-Water-Crop-Ambient Conditions

**DOI:** 10.3390/s18010053

**Published:** 2017-12-26

**Authors:** James Brinkhoff, John Hornbuckle, Thomas Dowling

**Affiliations:** 1 Centre for Regional and Rural Futures Deakin University, Hanwood 3217, New South Wales, Australia; j.hornbuckle@deakin.edu.au; 2 Goanna Telemetry Systems, Goondiwindi 4390, Queensland, Australia; tdowling@goannatelemetry.com.au

**Keywords:** water depth sensors, soil moisture sensors, temperature sensors, rice field monitoring, irrigation

## Abstract

Multisensor capacitance probes (MCPs) have traditionally been used for soil moisture monitoring and irrigation scheduling. This paper presents a new application of these probes, namely the simultaneous monitoring of ponded water level, soil moisture, and temperature profile, conditions which are particularly important for rice crops in temperate growing regions and for rice grown with prolonged periods of drying. WiFi-based loggers are used to concurrently collect the data from the MCPs and ultrasonic distance sensors (giving an independent reading of water depth). Models are fit to MCP water depth vs volumetric water content (VWC) characteristics from laboratory measurements, variability from probe-to-probe is assessed, and the methodology is verified using measurements from a rice field throughout a growing season. The root-mean-squared error of the water depth calculated from MCP VWC over the rice growing season was 6.6 mm. MCPs are used to simultaneously monitor ponded water depth, soil moisture content when ponded water is drained, and temperatures in root, water, crop and ambient zones. The insulation effect of ponded water against cold-temperature effects is demonstrated with low and high water levels. The developed approach offers advantages in gaining the full soil-plant-atmosphere continuum in a single robust sensor.

## 1. Introduction

Multisensor capacitance probes (MCPs) feature multiple sensors along a vertical tube [1]. Some have sensors that can be distributed along the plastic tube as needed, such as Sentek EnviroScan [2] probes. Others are sealed and have sensors fixed at regular intervals, for example 100 mm, as is the case for EnviroPro [3] probes. Each sensor in a probe is typically able to sense volumetric water content (VWC), temperature and conductivity. This makes them useful for characterizing those variables at multiple depths through the soil profile [4].

MCPs are typically used in irrigated crops, to monitor soil moisture status and schedule irrigations [5]. They are widely used in horticulture, cotton and cereal crops. By calculating the total water content, and checking for leeching beyond the root zone, optimal irrigation quantities can be determined [1]. Their characteristics with respect to temperature, salinity and soil type have been well studied, and calibrations developed to accurately determine volumetric water content from raw probe readings for numerous soil types [6,7]. Some probes, such as those used in this study, have calibrations for moisture variation with temperature and conductivity built in [3].

The demands of growing rice in temperate regions are quite different from those of other crops. Low temperatures at the microspore stage cause spikelet sterility [8], particularly for low temperatures around the rice plant panicle and to a lesser extent around the root zone. Deep ponded water has been used in temperate rice-growing regions to insulate against cold-temperature events [9]. However, in semi-arid regions, water is scarce, and ponded water leads to an increase in rice crop water use [10]. So careful management of water depth in rice paddies is critical to achieve environmental and productivity goals. This necessitates monitoring and management of water level, as well as temperatures at the root zone, in the water, at pannicle height and ambient. Many of these considerations, specifically for the Australian rice growing environment, are detailed in [11], where a water depth of 250–300 mm is recommended during the microspore growth phase to guard against cold-induced sterility.

Recently, techniques to minimise water use have been investigated, such as alternate wetting and drying (AWD) and delayed permanent water (DPW) [12,13]. Also, growing rice aerobically in temperate regions is gathering interest [12,14]. These developments require the monitoring of soil moisture to ensure sufficient water is available for rice plant growth during periods where the water is drained. Within Australia, water scarcity and increased competition for water is seeing many farmers actively look to dynamically manage water height for controlling the temperature the rice crop is exposed to during critical growth periods. This will aid in maximising yield and improving water use efficiency by maintaining high water levels only when required.

Various sensors can be used to automatically monitor these parameters (water depth, temperatures, soil moisture status). Capacitance-based fluid level sensors have been used in many applications, for example to measure fuel tank levels in [15]. An integrated soil moisture and water depth sensor for rice paddys based on a single capacitance sensor was presented in [16]. An array of sensors was used to monitor air and soil temperature, soil moisture and water height of rice fields in [17].

In this paper, we demonstrate the usefulness of MCPs as a robust option to simultaneously monitor all these parameters in a single probe. This provides advantages over the traditional method of using multiple sets of discrete sensors. Temperatures at multiple heights and volumetric water content (VWC) data is gathered by a single MCP. To provide an accurate estimation of water depth, the sensitivity to water depth within each of an MCP’s individual sensor’s 100 mm zone is characterized, and models are fit to the characteristics. Models are first developed for these 100 mm sensor zones, then these model parameters are used as initial conditions to fit global models covering the whole length of the MCPs.

WiFi-based loggers developed by the authors, named WiFields, were used to gather data from the sensors [18]. They were deployed in a rice field, simultaneously gathering data from EnviroPro MCPs, and MaxBotix MB7389 ultrasonic distance sensors. Comparisons between water depth measured using an ultrasonic distance sensor and a MCP are provided, as well as demonstration of the utility of gathering the temperature and soil moisture profile in fine (100 mm) increments.

## 2. Materials and Methods

### 2.1. WiFi-Based Data Loggers

WiFi-based agricultural data loggers (WiFields) were used to gather and upload sensor data. These loggers were developed by the authors, and make use of an Electric Imp (http://www.electricimp.com/) imp002 module which integrates a low-power microcontroller, WiFi radio and antenna. The loggers include common agriculture sensor interfaces such as SDI-12, UART and One-Wire. The loggers are programmed to periodically poll the sensors (typically once per hour), store sensor data, and if a WiFi connection to the Internet is available, upload all stored data to a cloud-based service, such as Google Sheets. The loggers then go into an ultra-low power sleep state until the next reading is due, saving power and ensuring battery operation over a whole cropping season. More detail on the WiField logger electronic design and software is available in [18].

### 2.2. Sensors

#### 2.2.1. Ultrasonic Distance Sensors

MaxBotix MB7389 ultrasonic distance sensors (https://www.maxbotix.com/Ultrasonic_Sensors/MB7389.htm) were used to provide an independent measure of water depth. They were positioned above the water surface, facing directly downwards. The water depth could then be determined by:

(1) $$d=d_{\mathrm{ground}}-d_{\mathrm{reading}}$$

In the equation, *d* is the water depth, $d_{\mathrm{ground}}$ is the distance between the sensor and the ground under the water, and $d_{\mathrm{reading}}$ is the distance reported by the sensor to the surface of the water, all in mm.

The MB7389 sensors were chosen for a number of reasons. They are robust and suitable for outdoor environments. They operate at 42 kHz, and have a narrow field-of-view, so there is less impact from interference from nearby plants or other objects. They include DSP to filter out noise from small reflections, so that the distance to the target with the largest acoustic return (in this case the water surface) is the one reported. They are low power, and operate from 3.3 V, which is the regulated supply voltage for many parts of the WiField logger circuitry. They were connected to the UART interface of the WiField loggers, providing readings with a resolution of 1 mm. The raw strings transmitted over UART were parsed by the logger code to determine $d_{\mathrm{reading}}$ as an integer, with units of mm.

**Figure 2:**
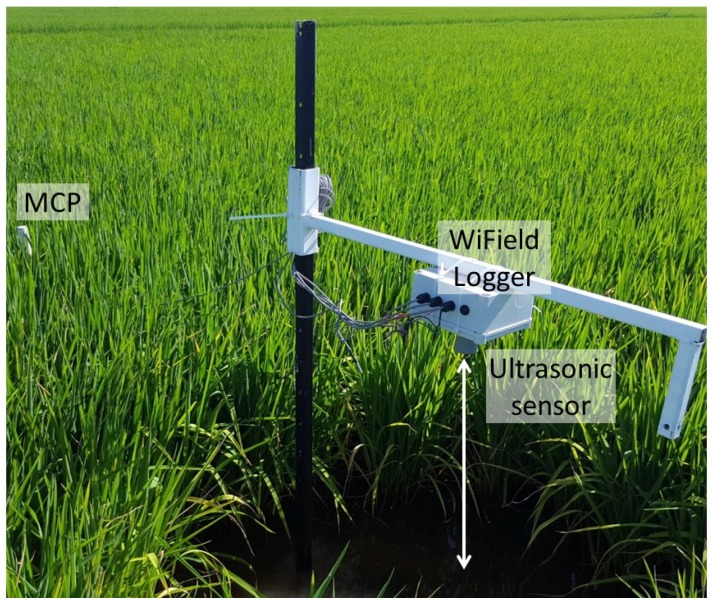
Photograph of a WiField data logger installed in a rice field with ultrasonic water level sensor, temperature sensors and MCP attached.

The ultrasonic sensors were used in laboratory characterization of the MCPs, and in field verification. For the field verification, the sensors were suspended above the ponded water of the rice crop as shown in Figure 2. Rice plants under the ultrasonic sensor were removed in order not to obscure the principal reflection from the water surface.

#### 2.2.2. Multisensor Capacitance Probes (MCPs)

The multisensor capacitance probes used were manufactured by EnviroPro [3]. The probes were connected to the SDI-12 interface of the WiFields. These MCPs are calibrated and sealed at the factory. They are designed to be used as soil moisture probes with a moisture, conductivity and temperature sensor at 100 mm intervals along the length of the probe. In this study, we used 8-sensor (800 mm long) and 12-sensor (1200 mm long) probes. The moisture readings are returned as volumetric water content (VWC) in m${}^{3}$/m${}^{3}$ as a percentage. This VWC is determined inside the probes from dielectric constant readings and is calibrated for a sandy soil. The user does not have access to the raw dielectric constant data, but calibration equations to scale the VWC to other soil types are provided by the manufacturer. The VWC is also calibrated against temperature and conductivity (salinity) variation inside the probes [3]. The moisture, temperature and conductivity readings returned over the SDI-12 interface were parsed by the logger and uploaded to the cloud-based Google Sheet as floating-point values. The spreadsheet is then further processed in Python to generate data directly useful to growers.

**Figure 1:**
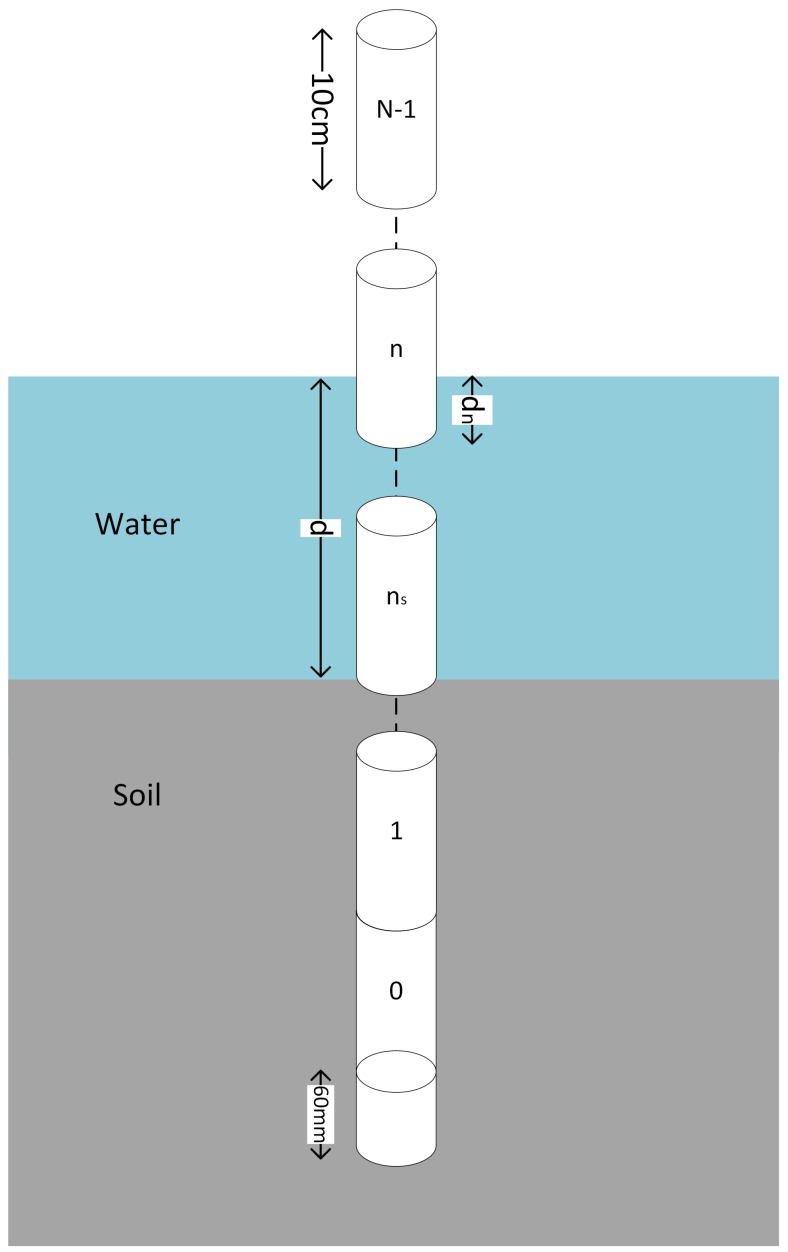
Diagram of a multiple-sensor capacitive soil moisture probe. The probe contains N−1 sensors, *d* is the total water depth in mm, $d_{n}$ is the water depth relative to the bottom of the *n*-th sensor and $n_{s}$ is the sensor sitting above soil level. Each of the sensors returns volumetric water content (VWC) (%), temperature (°C) and conductivity (dS/m) data.

A diagram of a probe is shown in Figure 1. The bottom 60 mm of the probes were found to be insensitive to moisture. For a probe with N sensors, the total probe length is N×100+60 mm. The bottom $n_{s}$ sensors are buried in the soil. The remaining sensors above the soil can be used to determine the level of the water. The water depth relative to the $n^{th}$ sensor is $d_{n}$ (with a range of 0–100 mm), and the total water depth in mm is

(2) $$d=\sum_{n_{s}}^{N-1}d_{n}$$

Figure 2 shows a photo of a WiField logger installed in a rice field, with connected ultrasonic and MCP sensors.

### 2.3. Models for Water Depth as a Function of the MCP VWC

The characteristics of the MCP VWC as a function of water depth were measured in a laboratory using a WiField logger. The probe was placed inside a 150 mm diameter PVC pipe. The ultrasonic sensor was used to measure the actual water depth. As the pipe was slowly filled with water, the water depth from the ultrasonic sensor, and the VWC from the MCP were logged frequently. The MCP measures the dielectric content of the surrounding medium, and calculates the VWC (assuming a sandy soil) for each of the 100 mm spaced sensors as a percentage ($\theta_{n_{\mathrm{raw}}}$). Only these VWC readings are available to the user (not the dielectric constant or frequency readings), so we developed the following methodology to calculate water depth from the available VWC data. The raw VWC of each sensor was normalized so that the VWC is between 0%–100% as follows:

(3) $$\theta_{n_{\mathrm{norm}}}=\frac{\theta_{n_{\mathrm{raw}}}-\theta_{{n_{\mathrm{raw}}}_{\min}}}{\theta_{{n_{\mathrm{raw}}}_{\max}}-\theta_{{n_{\mathrm{raw}}}_{\min}}}\times 100\;\left[\%\right]$$

If the *n*-th sensor is in air, the VWC $\theta_{n_{\mathrm{norm}}}$ will be zero, a fully submerged sensor will give of $\theta_{n_{\mathrm{norm}}}$ = 100%, and a partially-submerged sensor will be between 0%–100%. A simple linear model for water depth relative to each sensor is then:

(4) $$d_{n}=\frac{\theta_{n_{\mathrm{norm}}}}{100}\times d_{\mathrm{sensor}}\;\left[\mathrm{mm}\right]$$

where $d_{\mathrm{sensor}}$ is the spacing of the sensors along the probe, which is 100 mm in this case. These $d_{n}$ can then be summed to find the total water depth using (2). However, because of the in-built VWC calibrations and because the probe sensor characteristics vary over their 100 mm region of sensitivity, the curve relating the water depth to VWC is nonlinear. Each of the sensors is less sensitive at the 0 and 100 mm ends of their range than they are in the middle. Therefore, we evaluated a number of models that can better fit the depth vs VWC characteristic, which has an inverse-sigmoid shape.

The first was a general polynomial model of order *k*:

(5) $$d_{n}=\sum_{1}^{k}\alpha_{k}\theta_{n_{\mathrm{norm}}}^{k}$$

Next was a model using a tangent function, which has the desired inverse-sigmoid shape:

(6) $$d_{n}=\alpha_{1}\tan\left(\alpha_{2}\theta_{n_{\mathrm{norm}}}+\alpha_{3}\right)+\alpha_{4}$$

Knowing that $d_{n}=0$ when $\theta_{n_{\mathrm{norm}}}=0$, we can reduce the degrees of freedom of this tangent model to three:

(7) $$\alpha_{4}=-\alpha_{1}\tan\left(\alpha_{3}\right)$$

Various other probit functions were also evaluated, but they had similar characteristics and performance to the tangent model, so are not shown here.

The parameters of the above models were fit to depth vs VWC data using the optimizer functions of the SciPy Python library [19]. Reasonable initial guesses were provided for the tangent function parameters to improve convergence.

## 3. Results

### 3.1. MCP Laboratory Characterization

#### 3.1.1. Per-Sensor Model Fitting

The laboratory setup used the 150 mm diameter PVC pipe, with the MCP in the center and the ultrasonic sensor pointing down the pipe. Water was slowly added to the pipe, and the ultrasonic depth and MCP VWC logged continuously. If *d* is the water depth in the pipe from the ultrasonic depth sensor, and having determined the bottom 60 mm of the MCP is insensitive, the water depth relative to the $n^{th}$ sensor (being the currently partially submerged sensor, see Figure 1) of the MCP is

(8) $$d_{n}=\left(d-60\right)\;\mathrm{mod}\;100$$

**Figure 3:**
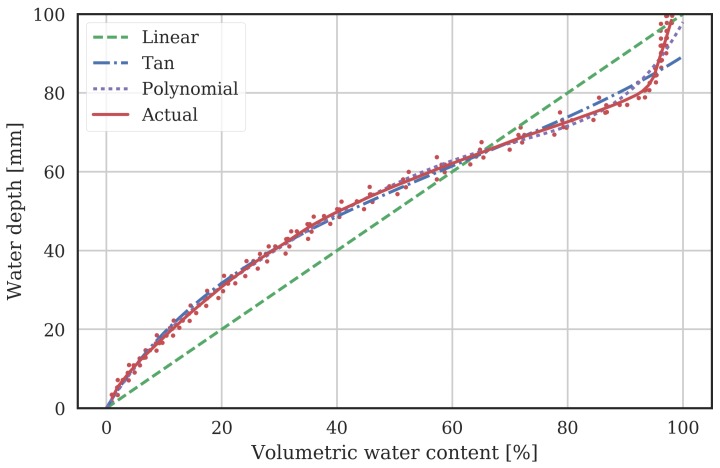
Water depth vs probe normalized VWC ($\theta_{n_{\mathrm{norm}}}$). Measured points from each sensor of the MCP are shown as red points, and the mean over all these sensors as a solid red line. Other lines indicate the water depth calculated from $\theta_{n_{\mathrm{norm}}}$ using various models.

We can then examine the characteristic of each of the 100 mm sensors individually. The PVC pipe was slowly filled to 660 mm, so the characteristics of 6 sensors could be determined. The modulo’d data is shown as points in Figure 3. The per sensor data was then interpolated and the mean over all sensors at each water depth was computed, and is shown as the solid red line in the figure. The resulting curve fits are shown in Figure 3, and the parameters of the models are shown in Table 1 in the “local” columns. The linear model (4) has up to 10 mm error over some $\theta_{n_{\mathrm{norm}}}$ values (i.e., at 20% and 90%). For the polynomial model (5), a number of polynomial orders were tried. Finally, a fifth-order polynomial was used, which gave reasonable accuracy while not oscillating between fit points, as higher-order polynomials tended to do.

#### 3.1.2. Whole Probe Model Fitting

**Figure 4:**
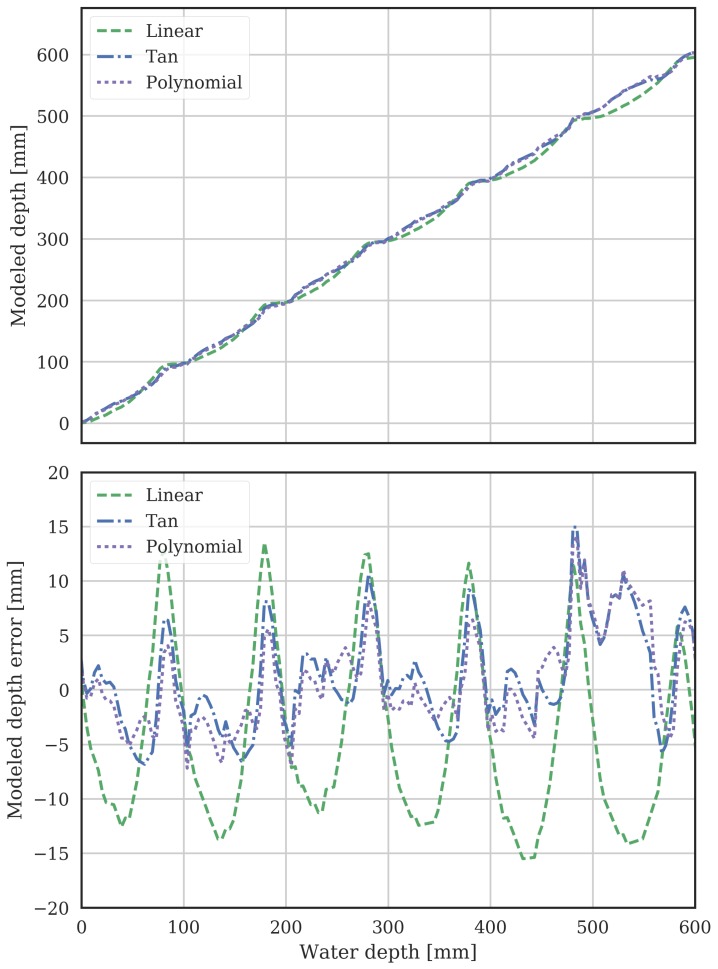
Water depth using the three models processing the characterized MCP’s VWC data, with actual water depth measured by the ultrasonic sensor on the x-axis. The bottom graph shows the error between the actual (ultrasonic) and modeled (MCP) depth.

Having fit local models to the mean of the MCP sensors characteristics, the obtained parameters were then used as initial conditions to optimize global models across the whole MCP length, which take account of overlap between the 100 mm sensitivity regions of the individual sensors. The global model depth is the sum of the individual sensor depths, from (2). The obtained global model parameters are also shown in Table 1. The modelled depth from VWC as a function of the actual water depth is shown in Figure 4. The ideal characteristic would be a straight line, with calculated depth from the MCP equalling measured depth from the ultrasonic sensor. The deviation between each of these models and the ideal straight line is shown in the bottom graph of Figure 4.

The root mean squared error (RMSE) is computed to give a quantitative assessment of the goodness-of-fit for each of the models. RMSE is defined as

(9) $$RMSE=\sqrt{\sum_{i=1}^{k}\frac{{\left(d_{i}-\hat{d_{i}}\right)}^{2}}{k}}$$

where $d_{i}$ is the actual water depth, $\hat{d_{i}}$ is the modeled depth (both in mm) and *k* is the number of readings. The RMSE is 9.9, 4.4 and 4.7 mm for the linear, polynomial and tangent models respectively. In the following results, only the tangent model results will be shown as it has similar accuracy to the polynomial model while requiring fewer parameters.

#### 3.1.3. Probe-to-Probe Variability

**Figure 5:**
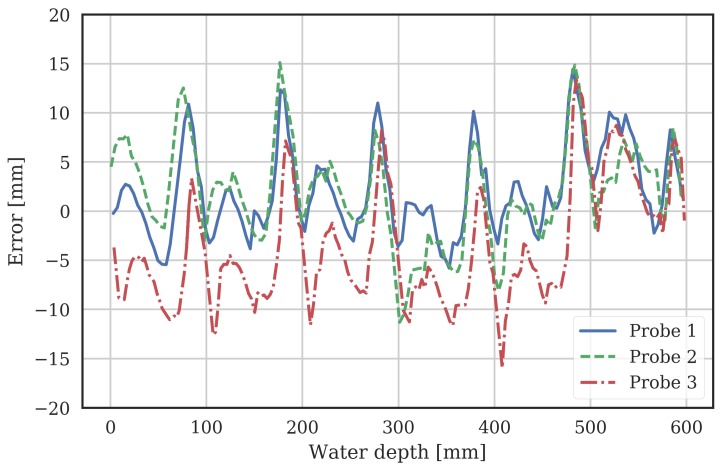
The error between actual (using the ultrasonic sensor) and calculated (using the VWC data from the MCP processed using the tangent model) water depth from 3 additional independent MCPs. Probes 1 and 2 are 12-sensor probes, and probe 3 is an 8-sensor probe.

The models above were extracted from a single 12-sensor probe in the laboratory. To assess variability from probe-to-probe, and to test the robustness of the characterization procedure, three additional probes were measured in the same laboratory setup. Two of these were 12-sensor probes, and one was an 8-sensor probe. The errors between measured water depth (from the ultrasonic sensor) and the MCP probe VWC data processed using the tangent model is shown in Figure 5. Note, the global model parameters in Table 1, which were extracted from the initial characterized probe, were used.

The RMSE (9) of the tangent model for the original probe the model was extracted from was 4.7 mm, as described in Section 3.1.2. The RMSE of the tangent model for these additional independent 3 probes was 4.9, 5.4 and 7mm for the two 12-sensor probes and 8-sensor probe respectively. This gives confidence that the model is well able to predict water level from the probe VWC readings, and the variation between probes is well controlled.

### 3.2. Rice Field Verification

In order to assess the usefulness of MCPs in a real rice growing situation, a WiField logger was installed in a field in Whitton, NSW, Australia (Figure 2). The rice variety was Topaz, the emergence date was 18 Novermber 2016, and harvest was on 12 May 2017. The soil type according the World Reference Base for Soil Resources [20] is Luvisol, and according to the local classification system is transitional red brown earth [21]. The WiField logger measured and uploaded data once per hour over the 2016–2017 rice growing season. The logger had the same sensors connected as those in the laboratory setup, a MaxBotix MB7389 ultrasonic sensor to measure ponded water depth and a 12-sensor EnviroPro soil moisture probe. In the example given, the top of the MCP was 700 mm above the soil, so water depths and above-ground temperatures to 700mm could be measured. The remaining 5 sensors were below the soil and could be used to monitor soil moisture and temperature to a depth of 500 mm. Generally, the rice crop root zone is within 200 mm of the soil surface, so an 8-sensor probe would be sufficient to measure both the rice root zone soil moisture and the ponded water depth. Since the usefulness of the technique was demonstrated in the 2016–2017 season, additional units are being used in additional rice fields in the current season.

**Figure 6:**
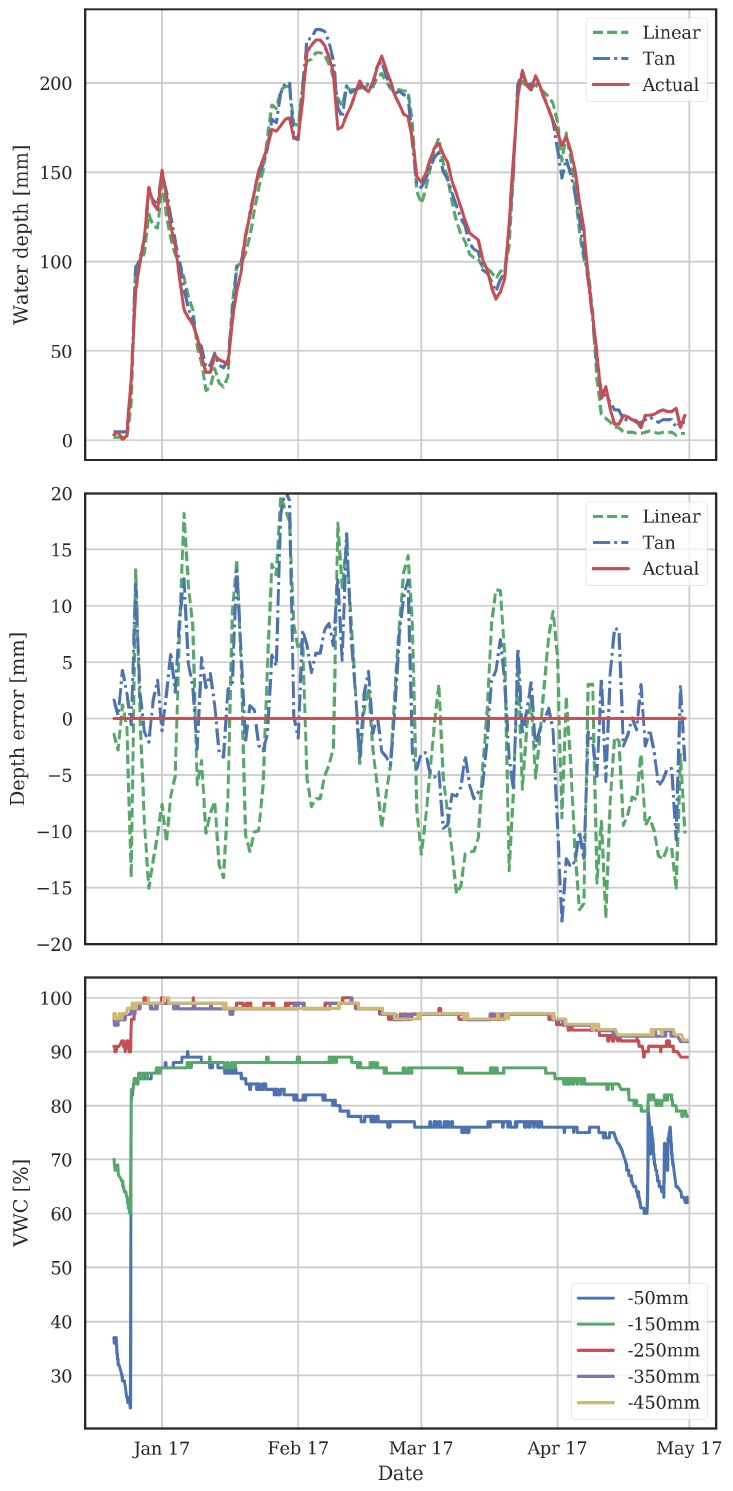
Measurements from a rice field over a growing season. The top graph shows the water depth from the ultrasonic sensor and from the MCP probe using the linear and tangent models. The middle graph shows the difference between the ultrasonic depth and depths calculated from the MCP readings. The bottom graph shows the soil moisture.

Figure 6 shows the water depth measured with the ultrasonic sensor, together with the water depth calculated from the MCP using the linear and tangent models. The RMSE over the season was 9.6 and 6.6 mm for the linear and tangent models respectively. The units functioned continuously over the season in the commercial farming situation and provided data in real time to the farmer that allowed him to actively adjust water depth on the fields based on the data. Note the farmer ensured the water was deep during the microspore growth period from late January to early February, to provide insulation against possible cold temperature events as recommended in [11].

**Figure 7:**
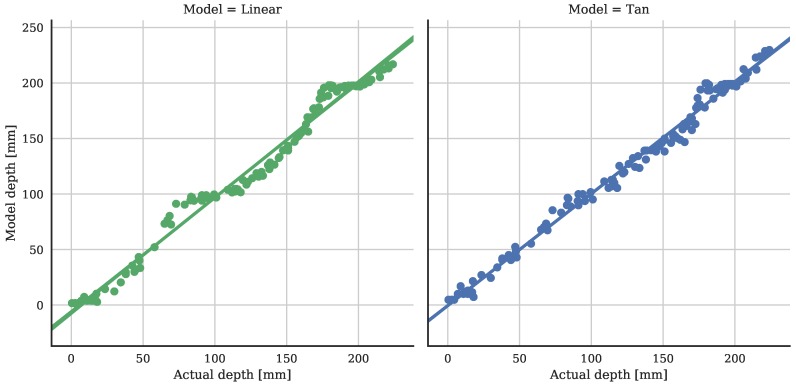
Regression plot of the modeled vs actual water depths from the rice field. The $R^{2}$ of the linear model is 0.985, and that of the tangent model is 0.991.

The field data is shown in Figure 7 as a regression plot, with the actual depth on the *x*-axis, and the modeled depth using the linear and tangent models on the *y*-axis. The equation of the linear model regression line is $d_{\mathrm{lin}}=1.036d-6.8$, with a coefficient of determination $R^{2}=0.985$, and *d* is the actual depth in mm. The equation of the tangent model regression line is $d_{\tan}=1.009d-0.56$, with $R^{2}=0.991$. These results show the tangent model from the MCP data gives a very good approximation to the actual water depth, with expected slope near 1 and intercept near 0.

#### Additional MCP Data

One advantage of using MCPs in this application is the range of useful data available. As well as water depth, which has been the focus of this paper, data on soil moisture and temperature at fine (100 mm) intervals is also obtained. The moisture data is shown in the lower graph of Figure 6. The soil never becomes unsaturated at depths below 200 mm. Early in the season before permanent ponded water is applied, the soil at 50 mm and 150 mm starts to dry out. It starts to dry again after the ponded water is drained in early-April. Some re-wetting of the soil at 50 mm due to rain events is evident in mid-April. Using the soil moisture information is particularly important in these early and late parts of the rice growing season to ensure the soil does not reach moisture stress levels, which would be detrimental to yield. The absolute VWC numbers depend on soil type and structure and varies with depth, as described in [22]. Calibration of these effects is beyond the scope of this paper but will be important areas of future study in using MCPs to schedule irrigations of rice fields with prolonged periods without ponded water. This is of particular relevance when managing newer water techniques such as alternate-wetting-drying (AWD) and delayed permanent water (DPW) as well as scheduling irrigation for newer aerobic rice varieties [12]. The variation of the VWC at 50 and 150 mm while permanent water is applied may be due to effects such as the growing roots affecting the air filled porosity of the soil.

**Figure 8:**
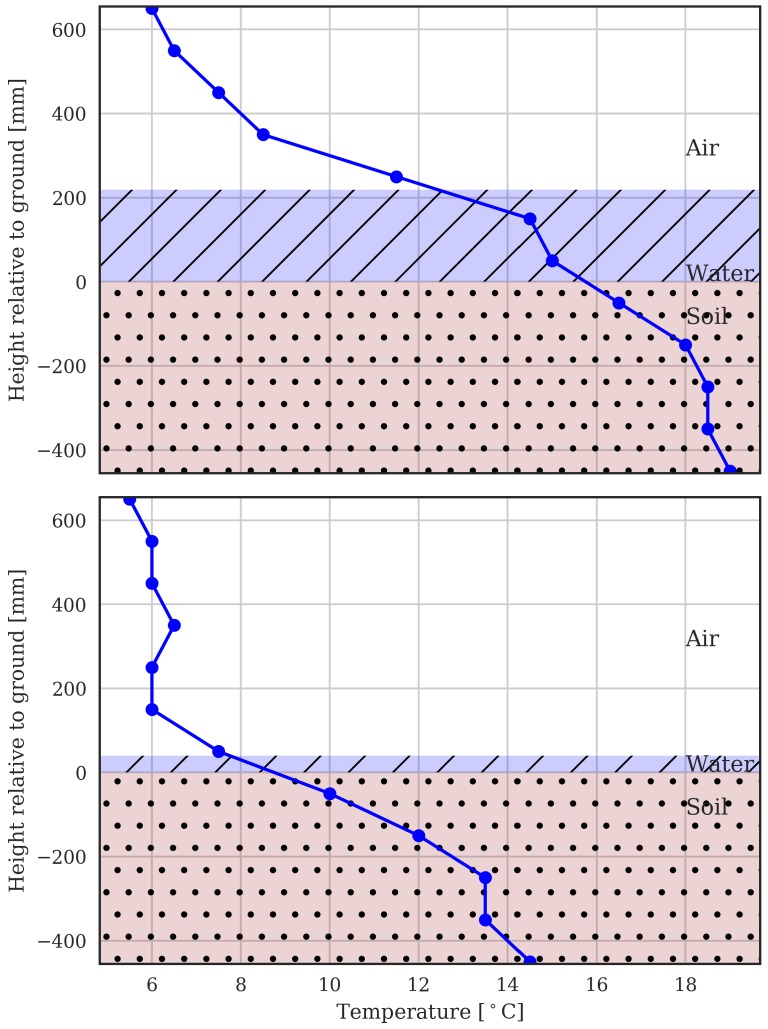
The temperature profile measured by the MCP during cold temperature events (20 February 2017 and 12 April 2017 6AM). The measured water depth is indicated with blue shading.

The temperature data from the rice field at two dates is shown in Figure 8. On both of these dates, there were signigicant cold events where the ambient temperature got as low as 6 ${}^{\circ }$C. The first date is 20 February 2017, which for a late-sown crop in NSW, Australia would fall close to the critical microspore phase. If the rice panicle was subject to the cold ambient temperature of 6 ${}^{\circ }$C, there would likely be a significant negative impact on yield. The second date is 12 April 2017, when there would not be such sensitivity to cold because the rice has passed the microspore phase. On the first date, the water was above 200 mm. It can be seen that this provided effective insulation against the cold temperatures at panicle height (around 200 mm), keeping the temperature there above 12 ${}^{\circ }$C. The water effectively maintains warmer temperatures above the surface of the water through its stored energy. In contrast, at the second date, no such insulation was provided as the water had been nearly drained. This provides a useful illustration of how temperature at multiple heights can be used to manage rice paddy water depth to effectively insulate the crop from cold temperatures. For temperate growing conditions with such cold events, recommended water heights to guard against cold-induced yield loss are given in [11]. It is anticipated simultaneously gathering water depth and the comprehensive temperature profiles that the MCP makes possible, will enable optimization and automation of water depth.

## 4. Discussion

In rice field monitoring applications, there is a need to monitor temperatures and soil moisture as well as water depth. Table 2 shows typical sensor types used to measure these parameters, and their relative cost, accuracy, robustness in field conditions, and typical interface used to connect the sensor to a data logger. Of course, there are a wide variety of manufacturers producing each of these sensors, so the table is only intended to be indicative of common examples.

Sensors that are typically used to monitor water depth include those using ultrasonic, pressure and capacitance measurements. Pressure sensors are accurate, but expensive. Ultrasonic sensors are subject to interference in open environments such as agricultural fields, particularly from insects, spiders and plants that grow into the field-of-view, so tend to not be robust in field environments and require regular maintenance. Many single capacitance sensors are not very accurate, being subject to variation with temperature and salinity, for example [23].

Soil moisture may be monitored with capacitance probes or gypsum blocks. The latter requires an AC resistance interface in order not to polarize the sensor. This interface is not available on many data loggers. It is usually desired to monitor moisture at multiple depths to gain an understanding of the moisture available throughout the root zone, so multiple sensors are needed.

Temperature sensors come in many types, with the digital DS18B20 sensors being quite common. For rice monitoring, multiple sensors are needed to determine soil, water, crop and ambient temperatures. It can be seen that simultaneously monitoring all the parameters of interest requires loggers with multiple interface types.

In view of the above, a MCP offers a unique solution. It measures many of the required environmental parameters in one robust unit with a single data interface. So multiple temperature sensors, a separate soil moisture sensor and water depth sensor are not required. This simplicity is attractive in agriculture, where reliability, robustness and lack of clutter are important considerations. For monitoring water level, MCPs offer a good trade-off between cost, accuracy and reliability. Though they are not inexpensive compared to some individual sensors, if the cost and interface requirements for a complete monitoring solution is considered (including soil moisture, water depth and multiple temperatures), their cost is not prohibitive relative to the benefits provided. It is also worth noting that many growers already own MCPs for other purposes. The MCPs can therefore be re-deployed for other uses during alternate seasons/years, so for example they could be used to monitor the water level in a rice field over the summer, and then the soil moisture in a wheat crop over the winter.

This study has developed techniques using the EnviroPro MCP. The same procedure of fitting models to the VWC vs water depth characteristic would be applicable to other MCPs too, provided their individual sensor sensitivity regions overlap, and that temperature and salinity variation is compensated, either by the probe (as is the case for the probes used in this study) or the user.

## Figures and Tables

**Table 1:** Curve fit parameters from MCP per-sensor VWC to water depth for each of the models. The local columns are the parameters extracted from the per-sensor data, and the global columns are the parameters optimized over the whole MCP.

Parameter	Tangent		Polynomial	
	Local	Global	Local	Global
$\alpha_{1}$	36.0	23.9	2.36	2.61
$\alpha_{2}$	16.8 × 10${}^{-3}$	22.7 × 10${}^{-3}$	63.5 × 10${}^{-3}$	−96.0 × 10${}^{-3}$
$\alpha_{3}$	−1.05	−1.12	1.44 × 10${}^{-3}$	2.46 × 10${}^{-3}$
$\alpha_{4}$			−17.1 × 10${}^{-6}$	−29.7 × 10${}^{-6}$
$\alpha_{5}$			76.0 × 10${}^{-9}$	131 × 10${}^{-9}$

**Table 2:** Comparison of using an MCP versus other sensor combinations to measure water depth, soil moisture and temperature.

Parameter	Sensor Type	Cost	Accuracy	Robustness	Typical Interface
Water depth	Pressure	$$$	+++	+++	40–20 mA
	Ultrasonic	$$	+	+	UART
	Capacitance	$$	+	++	Voltage
Soil moisture	Gypsum block	$	++	++	AC resistance
	Capacitive	$$	+	++	Voltage
Temperature	DS18B20	$	+	++	OneWire
All parameters	MCP	$$$	++	+++	SDI-12
